# Institutional barriers and professional integration of physicians with an Arab migration background in Germany: a qualitative study

**DOI:** 10.1186/s12992-026-01239-3

**Published:** 2026-10-08

**Authors:** Maria Noha El-Halabi, Jutta Hübner, Emadaldin Ahmadi

**Affiliations:** Klinik für Innere Medizin II, Jena University Hospital, Comprehensive Cancer Center Central Germany – Campus Jena, Am Klinikum 1, 07747 Jena, Germany

**Keywords:** Migrant physicians, Arab physicians, Professional integration, Germany, Healthcare system, Licensure, Working conditions, Discrimination, Intercultural competence, Qualitative research

## Abstract

**Background:**

Germany faces a persistent physicians shortage f, particularly in hospitals and rural regions. Migrant physicians, especially from Arab countries, represent a crucial component. However, their professional integration is shaped by complex licensure procedures, demanding working conditions, administrative burdens and sociocultural dynamics. While previous research has examined structural and regulatory barriers, limited empirical evidence exists on integration processes from the perspective of Arab physicians themselves.

**Purpose:**

This study aimed to explore the challenges and experiences of professional integration among physicians with an Arab migration background in the German healthcare system. Particular attention was paid to credential recognition, workplace conditions, language-related challenges, discrimination, cultural dynamics and patient-related barriers in order to identify institutional factors shaping integration processes.

**Methods:**

A qualitative research design was employed. Twenty physicians (16 men, 4 women) originating from Syria, Lebanon, Egypt and Yemen participated in semi-structured interviews conducted in Arabic or German. Participants were recruited through purposive and snowball sampling. Interviews lasted 45–60 min and were audio-recorded, transcribed verbatim and translated where necessary. Data were analyzed using reflexive thematic analysis to identify patterns and overarching themes across narratives. The analysis involved iterative coding and theme development, reflexive consideration of researchers’ interpretive roles and examination of divergent and contradictory cases. Theoretical saturation was assessed after approximately 18 interviews, with two additional interviews conducted to confirm that no new core topics emerged.

**Results:**

Five interconnected themes were identified. Participants described the German healthcare system as structurally reliable and well-resourced but characterized by high administrative workload, staff shortages and time pressure. Licensure procedures were experienced as lengthy, opaque and psychologically stressful. Administrative complexity and insufficient onboarding hindered early integration. Language barriers were primarily situational, relating to dialects and colloquial communication rather than medical terminology. Approximately half of participants reported experiences of discrimination, mainly from patients. Shared language and cultural background with Arab patients were perceived as professional resources that enhanced trust and communication. Participants also highlighted systemic barriers affecting patients with migration backgrounds, particularly language barriers and limited knowledge of the healthcare system. Participants additionally described an intermediary role in which their linguistic and cultural resources helped bridge communication and institutional gaps between patients with migration backgrounds and healthcare services. Across themes, professional integration emerged as a multidimensional and relational process shaped by institutional structures, workplace conditions, cultural dynamics and experiences of belonging and difference rather than solely as an individual adaptation process.

**Conclusion:**

Professional integration of Arab migrant physicians in Germany is strongly influenced by institutional structures, administrative processes and workplace conditions. While Germany offers robust structural prerequisites for medical practice, deficits in recognition procedures, onboarding, intercultural institutionalization and administrative support pose significant challenges. The findings suggest that integration should be approached as a reciprocal institutional process rather than solely as an adaptation task for migrant physicians. Structural reforms including streamlined licensure processes, structured induction programs, expanded interpreting services and systematic intercultural training are essential to support migrant physicians and strengthen the resilience and equity of the German healthcare system.

## Introduction

Germany has experienced an ongoing and critical shortage of physicians for many years, particularly in hospitals and rural areas. Projections indicate that the country will continue to face a shortfall of approximately 30,000 to 50,000 physicians between 2024 and 2040. This shortage reflects long-standing structural pressures within the German healthcare system, although the number of practicing physicians has shown some recent recovery [1]. Contributing factors include declining numbers of medical students, shortages of skilled personnel and persistent organizational deficits in hospitals [2].

Working conditions for hospital physicians in Germany have deteriorated in recent years. High workloads, long working hours and increasing administrative demands have substantially intensified occupational stress among hospital physicians. A recent survey found that nearly 60% of hospital physicians reported feeling overworked due to extended hours and excessive bureaucratic tasks [3]. Moreover, empirical studies have demonstrated significant associations between poor working conditions, effort–reward imbalances and both patients’ perceived quality of care and risks to patient safety [4]- [5]. Consequently, physician shortages cannot be viewed solely as a workforce issue but must be understood in relation to the structural and organizational environments in which physicians practice.

Migrant doctors form a critical component of the healthcare system in Germany. This has led to the consequential dependence on foreign physicians since there have been no viable training opportunities locally and due to the growing demand in healthcare [1]. A significant subgroup of the medical workforce is occupied by Arab migrant physicians (Syria, Egypt, Lebanon and Yemen). Their multilingual and intercultural competences can facilitate communication with culturally diverse patients and contribute to more culturally responsive healthcare [6].

The process of integrating into the workplace is more than just obtaining credentials and a job. Social sciences explain that integration is an ongoing multi-faceted process that includes gaining access to the institutional structure where one will work, developing a professional identity, obtaining the norms of the organization and establishing a sense of social belonging within the workplace environment [7].

Migrants have been very successful in integrating into the workplace; however, their success in becoming integrated depends upon not just the individual’s ability to perform, but also to the organisation’s culture and practices and to the broader society in which they live. Studies indicate that migrant physicians face multiple barriers to professional integration that extend beyond language proficiency. One of the most significant challenges is the recognition of foreign medical qualifications. Although formal procedures for obtaining medical licensure in Germany exist, the recognition process varies across federal states and is often characterized by prolonged processing times and administrative complexity [8]- [9]. These bureaucratic procedures can delay entry into the labor market and create uncertainty during the transition period, thereby complicating professional integration [10].

During the recognition process, migrant medical professionals usually receive a temporary or a limited license, so they cannot experience the benefits of having a stable job to practice and, in certain instances, practice lower than their qualifications [10]- [11]. There is evidence that the Syrian medical providers are stressed because of the delays, the lack of transparency and the instability of institutional support in the licensure process [12]. Integration barriers into workforce can cause delays in joining the workforce, which eventually results in more staff shortage.

Working conditions remain to be a challenge to many professionals even after they have attained their licensure. Cases of inadequate structure in postgraduate medical training programs in most professions including surgical specialties are documented. The problem of hard-working conditions among young doctors is slow to go because of the time, insufficient staffing, insufficient supervision and the rise in administrative burden that impedes the training process and negatively affect the hiring and retaining of young physicians [13]. The physicians who are migrants can experience even more levels of problems with the adaptation to these unfavorable working conditions since they simply do not have access to informal networks and mentoring systems.

Language-related barriers remain a significant issue in the daily clinical practice. It is also possible that due to differences in understanding medical terminology, regional dialects and unwritten communication standards, there can be breakdowns in communication between people. In some cases, it leads to the misinterpretation of professionals and, consequently, makes patients or fellow professionals think that they are incompetent [14]. Other researches also revealed that the unfamiliarity with the German healthcare system (legal systems, administrative processes and institutional levels) might be causing the uncertainty/stress among migrant physicians. These factors might also influence the perceived quality of care offered by those professionals to their patients [15].

Organizational socialization offers an insightful lens into how international-trained practitioners adjust to new and different working environments. By various means, both formal and informal, people obtain the requisite knowledge, abilities, values and behaviours necessary for inclusion within a professional community (or ‘work culture’). A review of prior studies demonstrates that the level of transparency in institutional procedures, the perceived degree of collegial assistance and the access to informal networks all significantly contribute to the facilitation of professional acclimatization amongst migrant health care professionals [16].

Besides the already mentioned problems of coping with the new work environment as a foreigner, there are other social integration problems (establishing relationships with co-workers). Differences in communication styles, power structures and values between one’s home culture and German culture can pose significant barriers to collaboration with other professionals. Thus, it will take much more acculturation and flexibility to succeed in integrating into such workplaces. Some direction can also be given to new immigrants through colleagues who are in a position to assist them, in particular, the former immigrated colleagues, to successfully navigate through this transition [17].

On the patient level, similar challenges may arise in intercultural communication. Studies have shown that different cultural contexts shape the expression of pain, the involvement of family members in medical decision-making and communication styles [18]. Consequently, misunderstandings may occur within the German healthcare system when culturally influenced expectations and behaviors are interpreted through a different normative framework. In addition, narrative and scoping reviews demonstrate that racism, discrimination and a lack of intercultural sensitivity within healthcare systems contribute to delayed treatment, barriers to access and undersupply among vulnerable populations [19]- [20].

Evidence from previous research further indicates that barriers to the professional integration of migrant physicians occur at both systemic and institutional levels in Germany. Nevertheless, little empirical research has examined these processes from the perspective of Arab physicians. In this study, professional integration is understood as the process through which migrant physicians become established within the professional, organizational and social structures of the host-country healthcare system. Professional integration extends beyond formal employment and includes access to licensure, participation in workplace routines, professional development opportunities, collegial relationships and recognition as legitimate members of the medical profession. This process is shaped by both systemic factors, such as healthcare regulations and licensure procedures and institutional factors, including workplace cultures, onboarding practices, supervision and organizational support structures. Against this background, the present study explores how physicians with an Arab migration background experience professional integration into the German healthcare system and how systemic and institutional factors facilitate or hinder this process. Particular attention is paid to licensure procedures, workplace conditions, language-related challenges, discrimination, cultural dynamics and physicians’ perceptions of barriers affecting patients with migration backgrounds. The study further seeks to identify institutional support strategies that may facilitate sustainable professional integration.

## Methods

### Research design

This study employed a qualitative research design to explore the challenges and experiences of professional integration among physicians with an Arab migration background in the German healthcare system. A qualitative approach was selected to capture participants’ subjective perspectives and to gain an in-depth understanding of how institutional structures, professional practices and social contexts shape integration processes [21]. The study focused on identifying patterns, contrasts and underlying mechanisms across individual narratives. Data were collected through semi-structured interviews, which provided a flexible framework for exploring participants’ experiences while ensuring that key topics relevant to professional integration were addressed consistently across interviews.

### Sampling and participants

Recruitment to select physicians of Arab migration background practicing medicine in Germany or pursuing the licensure process was done through purposive sampling [22]. The inclusion criteria were (a) medical degree received either in an Arab country or in Germany, (b) having an Arab migration background and (c) current participation in the German healthcare system. The Final sample size was 20 physicians (16 men, 4 women) originating from Syria (*n* = 12), Egypt (*n* = 3), Lebanon (*n* = 3) and Yemen (*n* = 2). These countries were not predefined as selection criteria. Rather, they emerged through the recruitment process based on the study’s inclusion and exclusion criteria and the voluntary participation of eligible physicians. Exclusion criteria included physicians who had not yet entered the German healthcare system, individuals without a medical degree, physicians who come from countries where Arabic is not the official language and physicians who have no experience in either the German healthcare system or that of their home country. No otherwise eligible participants were excluded due to language barriers, as interviews were offered in both Arabic and German according to participant preference. The sample was purposively composed to capture variation in age, medical specialization, place of medical education, year of licensure application and workplace environment. The final sample comprised 20 physicians, 16 men (80%) and 4 women (20%), with ages ranging from 29 to 64 years (mean = 38.6 years; median = 34.5 years). The gender distribution therefore reflects an overrepresentation of male physicians, which should be considered when interpreting the findings. The sample included physicians who had completed their medical education either in Germany or abroad, allowing experiences of professional integration to be considered across different educational backgrounds.

Place of medical education may also have shaped participants’ experiences of professional integration. Physicians trained in Germany may have entered the healthcare system with greater familiarity with its institutional procedures, professional norms, communication practices and workplace expectations, whereas those trained abroad may have had to acquire this contextual knowledge after migration. Previous research similarly suggests that internationally trained physicians may require additional time and support to adapt to local professional and organizational practices [14, 23]. However, because our study was not designed to compare German-trained and internationally trained physicians systematically, these differences should be interpreted as contextual influences rather than as evidence of distinct integration trajectories. The demographic and professional variables are offered in Table 1.

**Table 1:** Demographic and professional characteristics of participants (N = 20)

**Variable**	***n***	**%**		***n***	**%**
**Gender**			**Languages Spoken**		
Male	16	80	Arabic, German	3	15
Female	4	20	Arabic, German, English	14	70
**Country of origin**			Arabic, German, English, French	3	15
Syria	12	60	**Years Since Application for Medical Licensure in Germany (range: 3–43)**		
Lebanon	3	15	< 10	16	80
Egypt	3	15	10–15	1	5
Yemen	2	10	> 15	3	15
**Country of medical education**			**Medical specialty group**		
Germany	5	25	Surgical specialties^a^	12	60
Syria	11	55	Medical specialties^b^	6	30
Egypt	3	15	Diagnostic specialties^c^	2	10
Lebanon	1	5	**Living situation**		
**Current workplace**			With family	8	40
Hospital	16	80	With spouse	5	25
Private practice (owner)	4	20	Alone	7	35
**Years living in Germany**			**Duration of Medical Practice in Germany**		
< 10	16	80	< 10	17	85
10–15	1	5	10–15	0	0
> 15	3	15	> 15	3	15
	**M**	**SD**			
**Age (years) range: 29–64**	38.6	10.1			

^a^ Surgical specialties: General Surgery, Plastic Surgery, Gynecology, Orthopedic Trauma Surgery, Neurosurgery, Ear-Nose-Throat, Visceral Surgery, Vascular Surgery, Urology

^b^ Medical specialties: Cardiology, Internal Medicine, Neurology, Geriatrics, Psychiatry

^c^ Diagnostic specialties: Radiology

### Data collection

Participants were recruited through Facebook groups and snowball sampling via referrals from previously interviewed physicians [24]. Data were collected between April 2023 and December 2023 using semi-structured interviews [21]- [22, 25]. The interview design drew on the principles of qualitative interviewing described by Kvale and Brinkmann [25], with emphasis on openness to participants lived experiences and the dynamic, co-constructive nature of the interview process.

Interviews were conducted either in person or by telephone, depending on participant preference and logistical considerations. Although telephone interviews can facilitate participation across geographical distances, they may limit access to non-verbal communication cues and reduce opportunities for establishing rapport compared with face-to-face interviews. Consequently, certain nuances of participants’ experiences may not have been fully captured.

An interview guide was developed based on the study objectives and existing literature on migrant physicians, professional integration and organizational socialization [16]- [17, 26]. The interview guide was constructed following the systematic approach outlined by Kallio et al. [26], which provides a validated framework for developing semi-structured interview guides in qualitative research.

The guide consisted of questions asking participants about their time migrating to and integrating into Germany as professional people, as well as their experiences with the German healthcare system, challenges in licensing or getting work in their professions because of the language barrier and administrative processes, how they interact within their workplaces and culture and how these same challenges have had an impact on the care that they provide to patients with a migration history. In keeping with the reflexive thematic analysis methodology, the interview guide was not a rigid questionnaire, but rather a flexible framework that was adjusted to allow for the participant to elaborate on the topics they thought were important. This made it easy to explore any new topics that may arise from discussing with the participants their experiences in migrating and integrating into Germany professionally. The guide was iteratively refined during data collection and preliminary analysis to incorporate emerging topics [27]. The interview guide was designed to encourage broad exploration of participants’ experiences rather than to predetermine the final thematic structure. Not all interview topics developed into overarching themes, while several themes integrated insights across multiple interview domains.

Interviews were conducted in Arabic or German, according to participant preference and lasted approximately 45–60 min. All interviews were audio-recorded with consent and transcribed verbatim. Arabic-language interviews were translated into German by a bilingual member of the research team who is fluent in both languages and familiar with the cultural context of the participants. A formal forward–backward translation procedure was not conducted because the analysis was performed using the German transcripts. No transcripts were translated into English during the analytical phase. Quotations presented in the manuscript were translated by the members of the research team into English only for publication purposes.

### Data analysis

Data were analyzed using reflexive thematic analysis following the approach described by Braun and Clarke [28]- [29]. As a research team, we approached the analysis as an active and situated interpretive process, acknowledging that knowledge is co-constructed through the researcher’s engagement with the data rather than passively discovered within it. This reflexive orientation required continuous attention to the researchers’ positionality, assumptions and interpretive choices throughout the analytic process.

Transcripts were read repeatedly to achieve immersion and familiarity with the data. Initial impressions were documented in analytic memos. Open, inductive coding was conducted to identify meaningful text segments related to institutional processes, professional experiences and perceived barriers and opportunities. Codes were compared across interviews and grouped into preliminary themes.

The analysis was conducted as an interpretive and reflexive process rather than as a procedure for categorizing participants’ responses into predetermined content areas. Following Braun and Clarke’s reflexive thematic analysis approach, codes were examined in relation to one another and developed into candidate themes representing broader patterns of shared meaning. Crucially, the research team’s disciplinary backgrounds in medical sociology, migration studies and health services research informed but did not predetermine the analytic process. Throughout the analysis, the team engaged in critical reflexive practice by interrogating how their own positions, prior knowledge and disciplinary framings shaped what was noticed, how codes were applied and which interpretations were privileged.

Candidate themes were repeatedly reviewed against the coded extracts and the dataset as a whole, with attention to their internal coherence, distinctiveness and relevance to the research questions. Themes were subsequently refined and defined in terms of their central organizing concepts rather than being retained simply because they represented frequently discussed topics. Saturation was conceptualized as the development of a sufficiently rich and complex thematic account, rather than as the mere exhaustion of new codes.

Coding and theme development were recursive rather than linear, with the researchers moving iteratively between coded extracts, the dataset as a whole and developing interpretations.

Theoretical thematic saturation was assumed when additional interviews no longer generated substantially new themes but instead confirmed and further differentiated existing categories [30]. This approach to saturation is consistent with the reflexive thematic analysis framework, in which the goal is the development of a comprehensive and nuanced thematic account rather than the enumeration of all possible data variations. Saturation was therefore assessed in terms of the depth, coherence and explanatory power of the developing themes, rather than as a fixed numerical endpoint.

After approximately 18 interviews, no new core topics emerged. Two additional interviews were conducted to confirm saturation across different medical specialties and regional contexts. The saturation assessment was used as a criterion for determining the adequacy of the dataset rather than as a substitute for the reflexive process of theme development and interpretation.

Two researchers independently participated in coding and theme development. Regular analytical discussions were held to reflect on coding decisions, resolve discrepancies and consider alternative interpretations. These discussions explicitly addressed how the researchers’ preconceptions and disciplinary perspectives could shape analytic outcomes and how alternative interpretations might be considered. Deviant or contradictory cases were deliberately examined to enhance analytical depth. Such cases were treated as opportunities to challenge and refine the developing themes, rather than as anomalies to be reconciled with predetermined categories. By engaging with negative or disconfirming instances, the research team was able to strengthen the conceptual boundaries and interpretive depth of the themes. These discussions also provided opportunities for reflexive consideration of how the researchers’ disciplinary backgrounds, knowledge of migration and professional integration and expectations concerning migrant physicians could influence interpretation. Rather than treating coding differences as evidence of a single objectively correct interpretation, the researchers used these discussions to critically examine alternative understandings and refine the developing themes.

According to Braun and Clarke’s method of reflexive thematic analysis, themes were treated not as summaries of interview topics but rather as ways of interpreting patterns of shared meaning, which were created through the active process of working with data. This framing underscores that the resulting themes are interpretive constructions, situated within specific analytic and disciplinary contexts, rather than neutral descriptions of participant responses.

In keeping with the reflexive orientation of the analysis, the interpretation emphasized the relational and contextual dimensions of professional integration, including how institutional structures, interpersonal dynamics and individual agency intersected to shape participants’ experiences.

The resulting themes are therefore analytic representations of the experiences of participants, not merely direct categorizations of domains on the interview guide.

### Trustworthiness and rigor

Several strategies were employed to ensure the trustworthiness of the study [31]. Credibility was supported through prolonged engagement with the data, iterative analysis and the use of verbatim quotations in reporting. Dependability was strengthened through systematic documentation of analytic procedures. Confirmability was promoted through reflexive memo writing and team-based discussions of analytic decisions. Detailed descriptions of participants and institutional contexts facilitate assessment of the transferability of findings to comparable settings.

### Ethical considerations

Participants were informed verbally about the study objectives, procedures, confidentiality measures and their rights as participants, including the right to withdraw from the study at any time without consequences. Verbal informed consent was obtained prior to each interview. This form of consent was considered appropriate given the low-risk nature of the study, which focused on healthcare professionals’ experiences in their professional roles rather than on sensitive personal or health-related information. In addition, verbal consent supported participant anonymity and reduced administrative burden. All personal data were anonymized during transcription. Audio recordings and transcripts were stored securely on password-protected systems and were accessible only to members of the research team. According to the regulations of the Ethics Committee of Jena University Hospital, formal ethical approval was not required for interviews conducted with healthcare professionals about their professional experiences.

### Funding declaration

This research did not receive any funding.

## Results

The results are organized around five interconnected themes that together illustrate professional integration as a progressive and overlapping process. Rather than presenting isolated challenges, the themes are presented in a sequence that reflects participants’ experiential trajectory: from initial entry into the profession (Theme 1), through familiarization with institutional structures (Theme 2) and the broader healthcare system (Theme 3), to the development of intercultural competence (Theme 4) and the emergence of a mediating role between healthcare institutions and patients with migration backgrounds (Theme 5).

The themes were developed through an iterative and reflexive engagement with the data rather than being derived directly from the interview guide. Initial codes were examined in relation to one another and developed into candidate themes representing broader patterns of shared meaning. Candidate themes were repeatedly reviewed against coded extracts and the dataset as a whole and were refined according to their internal coherence, distinctiveness and relevance to the research questions. The final themes therefore represent interpretive patterns of shared meaning across participants’ accounts rather than direct categorizations of the topics addressed during the interviews.

1. Seeking Recognition and Professional Legitimacy

This theme captures how entry into the German healthcare system was experienced as a prolonged process of institutional uncertainty. Participants described licensure and employment procedures as shaping their earliest experiences of professional integration, belonging, recognition and legitimacy within the healthcare system.

Participants’ accounts further indicated that these challenges were experienced as part of a broader process of establishing professional legitimacy within the German healthcare system. Rather than representing isolated administrative obstacles, prolonged recognition procedures, uncertainty regarding requirements and difficulties obtaining employment shaped participants’ early perceptions of their professional position and belonging within the system.

I had to submit the same original documents multiple times, including my police clearance certificate. It took me three months to find a chief physician willing to hire me without full licensure and the entire recognition process lasted almost two years. (P15)

(…) They kept asking for more documents. I would submit one and two weeks later they would request another one. It was constant back and forth with the paperwork and the recognition alone took one and a half years. (P20)

Having established the prolonged process of seeking professional legitimacy, the following theme examines how participants subsequently learned to navigate the institutional complexity they encountered upon entering the German healthcare system.

2. Learning to Navigate Institutional Complexity

This theme reflects the process through which physicians learned to navigate unfamiliar institutional structures. Participants’ experiences highlighted that successful integration depended not only on individual competence but also on organizational support, transparency and access to informal learning opportunities.

All participants described difficulties navigating administrative processes, including applications, documentation, rehabilitation referrals and legal forms. These challenges were most pronounced during early employment stages and were attributed to insufficient institutional onboarding rather than individual deficits.

I wasn’t well trained… I would definitely have liked better training. (P8)

Language difficulties were concentrated in early integration phases and related primarily to dialects and colloquial patient communication rather than medical terminology. Linguistic insecurity was often misinterpreted as professional insecurity, though this perception diminished with increasing routine. Collegial support was crucial in overcoming language-related challenges. Medical German was not the main problem.

I don’t really have language barriers anymore. Back then, the main challenge was strong regional dialects. You only learn medical German, not the individual dialects. Over time, you learn the dialect of the federal state where you work and live. (P17)

If I realize that the dialect is causing the language barrier, I take more time and ask the patient to explain it more slowly and more clearly. (P13)

Approximately half of participants reported experiences of discrimination, mainly from patients. These ranged from subtle mistrust to open rejection of treatment.

Do you have any allergies? – No, but I have an allergy to people who look like you. (P4)

Marked regional variation was observed. Participants developed pragmatic coping strategies such as ignoring minor incidents, redirecting conversations, or involving supervisors.

Taken together, these accounts indicate that professional integration involved more than learning formal procedures within the German healthcare system. Participants also had to navigate linguistic demands and interpersonal experiences that shaped their sense of recognition, belonging and professional security. Institutional complexity was therefore experienced not only through administrative processes but also through everyday interactions in which language, cultural difference and experiences of racism could influence participants’ ability to participate fully in professional life.

Building on these experiences of navigating institutional complexity, the following theme examines how participants evaluated the broader structural characteristics of the German healthcare system in comparison to their countries of origin.

3. Practicing Within a Reliable but Pressured System

This theme captures how participants evaluated the structural foundations of professional practice in Germany. While the healthcare system was viewed as providing reliable and high-quality conditions for medical work, participants simultaneously described organizational pressures that constrained their professional autonomy and everyday practice. The theme therefore reflects an underlying tension between structural opportunity and workplace strain. Participants frequently compared the German healthcare system with those of Syria, Lebanon, Egypt and Yemen as a way of contextualizing their professional experiences.

The German system was consistently described as structurally reliable, well resourced and highly standardized. Participants emphasized stable emergency services, widespread availability of diagnostics and therapies, digital documentation, preventive services and compulsory health insurance as fundamental prerequisites for medical practice. These features contrasted sharply with experiences of resource scarcity, political instability and interrupted care in countries of origin. Importantly, these advantages were viewed less as sources of daily job satisfaction and more as baseline conditions that enable professional practice.

Regardless of whether you have private or statutory insurance, every patient receives the same high-quality treatment. (P19)

Disadvantages of the German system were primarily associated with working conditions rather than clinical standards. Three interrelated patterns emerged: high bureaucracy, staff shortages and time pressure. Documentation, billing and reporting obligations were perceived as excessive and as limiting time for direct patient contact.

I have more contact with my work computer than with my patients. (P19)

Staff shortages intensified workloads through overtime, additional shifts and limited recovery periods.

Due to the shortage of doctors, I have to take on more and more shifts per month. (P16)

Participants emphasized that these challenges were experienced by both migrant and German-trained physicians. However, unfamiliarity with administrative procedures and institutional structures made the early integration phase particularly demanding for migrant doctors.

These accounts reveal a central tension between the structural reliability of the German healthcare system and the pressures associated with everyday clinical work. Access to resources, infrastructure and organized professional structures created important opportunities for effective practice, while workload, staffing pressures, time constraints and organizational demands could limit the extent to which these structural advantages were experienced as supportive in daily practice. The theme therefore reflects the coexistence of institutional reliability and occupational strain within participants’ professional experiences.

Beyond the structural and organizational dimensions of professional practice, participants’ cultural backgrounds also played a significant role in shaping their clinical experiences, as examined in the following theme.

4. Intercultural Competence as Resource and Difference

This theme illustrates how cultural background functioned simultaneously as a professional resource and a source of difference. Participants described intercultural competence as an important asset while also encountering situations in which cultural and religious identities became visible and occasionally problematized within workplace interactions.

Shared language and cultural knowledge with Arab patients were widely described as professional assets that facilitated trust, nuanced interpretation of symptoms and communication in emotionally charged situations. Participants emphasized adaptation to German cultural norms while retaining intercultural competence.

Doctors with a migration background often have better cultural competence, which can help them to better understand the needs and expectations of their patients with a similar migration background. (P5)

Implicit cultural insensitivities were occasionally reported, particularly regarding religious practices such as fasting during Ramadan.

They don’t understand that Muslim doctors can work just as efficiently when fasting. (P11)

Despite these experiences, most participants described supportive professional relationships and emphasized reciprocal cultural consideration, such as working on Christian holidays.

While cultural preferences (e.g., physician gender, family presence) were often accommodated in practice, respondents noted informal devaluation of such expectations within teams. A lack of institutional guidelines for managing cultural expectations was highlighted, alongside calls for intercultural training.

Taken together, these accounts suggest that cultural difference was not experienced as inherently advantageous or disadvantageous. Rather, its meaning was context dependent: cultural and linguistic knowledge could function as a professional resource when supporting communication and patient care, while the visibility of cultural or religious practices could also expose participants to misunderstanding, stereotyping, or interpersonal tension within the workplace. Intercultural competence therefore operated simultaneously as a professional resource and as a dimension through which difference could become visible and consequential.

The cultural and linguistic resources that shaped participants’ professional identities within the workplace also informed their interactions with patients, particularly those with migration backgrounds, as examined in the following theme.

5. Acting as Cultural and Institutional Mediators

This theme highlights how participants frequently occupied an intermediary position between healthcare institutions and patients with migration backgrounds. Their narratives revealed an often-unrecognized mediating role in which they translated not only language but also institutional expectations, administrative procedures and healthcare practices. Through their shared linguistic and cultural knowledge, participants helped bridge gaps between patients and healthcare organizations, thereby facilitating access, understanding and continuity of care.

Language barriers were identified as a primary obstacle for patients, affecting history-taking, informed consent and understanding of treatment. Professional interpreters were described as optimal but inconsistently available. In their absence, participants relied on multilingual colleagues, digital translation tools, or relatives.

Hospitals should provide interpreter services to ensure that patients who do not speak the language receive appropriate medical care. Too often, family members are used as interpreters, even though they may not be fully proficient in German, which can lead to the communication of misinformation. (P12)

Understanding referral pathways and the admission system, which is managed by general practitioners, posed major challenges for newly arrived patients. Participants reported spending approximately 10–15 min per consultation explaining system structures, depending on workload. Participants frequently described taking on an additional mediating role when caring for patients with migration backgrounds. Beyond their clinical responsibilities, they often explained healthcare procedures, clarified administrative requirements and facilitated communication between patients and healthcare institutions. Shared linguistic and cultural knowledge enabled them to bridge gaps in understanding and support patients in navigating unfamiliar healthcare structures.

Communication barriers sometimes resulted in shorter consultations or impatience under high time pressure. These differences were attributed to structural constraints rather than discriminatory intent.

Normally, you need five minutes per patient… but that’s not enough for patients with a language barrier. (P9)

Arabic-speaking patients were perceived as feeling more comfortable with linguistically and culturally concordant physicians.

Taken together, these accounts indicate that migrant physicians frequently assumed a mediating role extending beyond their formal clinical responsibilities. Their linguistic and cultural resources enabled them to bridge communication and institutional gaps for patients with migration backgrounds, while their familiarity with different healthcare expectations allowed them to support patients in navigating clinical encounters. At the same time, this additional mediating role created demands on consultation time and professional workload, highlighting the dual significance of this role as both a resource for patient care and an additional professional responsibility.

## Discussion

This study explored the professional integration experiences of physicians with an Arab migration background in the German healthcare system from their own perspective. The collective themes of a reflexive thematic analysis identified that professional integration is best conceptualized as a process of negotiating one’s institutional belonging within the context of an intricate and multidimensional healthcare system. Throughout this analysis, the individual themes did not represent discrete barriers or facilitators, but were indicative of a shared pattern where physicians routinely interacted with their organizational framework, administrative and operational systems, workplace cultures and the broader societal expectations of them. Therefore, professional integration also became evident as an institutionally mediated and relational process rather than simply an individual accomplishment. Using a qualitative design and reflexive thematic analysis, the findings demonstrate that professional integration is a structurally shaped process influenced by institutional arrangements, working conditions, administrative procedures and sociocultural dynamics. Across all themes, participants described integration not primarily as an individual adaptation task but as a process strongly shaped by systemic and institutional conditions. Although Germany was perceived as offering strong structural prerequisites for high-quality medical practice, substantial barriers persisted during licensure, early employment and everyday clinical work. In particular, shortcomings in onboarding, administrative support and intercultural institutionalization emerged as recurring challenges across participants’ narratives.

The findings may also be interpreted through the lens of organizational socialization and institutional belonging. Participants’ experiences suggest that integration was not solely determined by individual language proficiency or professional competence but was strongly influenced by opportunities to understand institutional norms, navigate administrative structures and establish a sense of legitimacy within professional settings. This interpretation aligns with broader social science perspectives that conceptualize integration as a relational and context-dependent process [32].

A central finding concerns the ambivalent perception of the German healthcare system. Consistent with previous research, participants emphasized structural reliability, standardized diagnostics and therapies and universal health insurance as major strengths [1]- [2]. These features contrast sharply with experiences of resource scarcity, political instability and disrupted care in several Arab countries. At the same time, disadvantages were primarily located in working conditions rather than clinical standards. High administrative workload, staff shortages and time pressure emerged as dominant stressors, reflecting findings from German and international studies demonstrating associations between unfavorable working conditions, effort–reward imbalance and reduced perceived quality of care [3]- [5]. Importantly, participants interpreted these challenges as system-level issues affecting all physicians, while noting that limited system knowledge and bureaucratic unfamiliarity made early integration particularly difficult for migrant doctors. This highlights that migration status interacts with existing structural problems rather than constituting an isolated source of disadvantage.

The findings further underscore that administrative complexity and inconsistent onboarding constitute major institutional barriers. Participants reported difficulties navigating documentation requirements, applications and legal procedures, especially during early employment. These experiences align with studies documenting long recognition processes, regional variation and limited transparency in licensure procedures for foreign-trained physicians [8]- [11]. Qualitative evidence from Syrian physicians similarly describes prolonged waiting periods and uncertainty during recognition, contributing to psychological distress and delayed workforce entry [12]. Together, these findings suggest that streamlining administrative procedures and strengthening structured induction programs may represent key leverage points for improving professional integration.

Language emerged as a situational rather than permanent barrier. Participants distinguished between medical terminology, which was acquired relatively quickly and context-specific challenges such as dialects and colloquial patient communication. This differentiation mirrors previous qualitative studies showing that communication difficulties often relate to pragmatic language use rather than formal proficiency [14]- [15]. Misinterpretation of linguistic insecurity as professional incompetence in early stages highlights the importance of supportive workplace cultures and mentoring structures. Collegial assistance played a central role in overcoming language-related challenges, underscoring the significance of informal support networks in integration processes.

In addition to language-related aspects of professional integration, participants’ narratives also pointed to concerns regarding the adequacy of clinical training and practical preparedness. Our findings indirectly support concerns raised in previous research regarding insufficient clinical training, particularly in surgical contexts [13]. Although surgical training was not examined as a primary outcome, reported dissatisfaction with technical and organizational aspects of care suggests that gaps in training and preparedness may continue to influence patient experiences.

### Racism, othering and coping strategies

A particularly important finding concerns the interplay between experiences of racism, cultural dynamics and processes of othering reported by participants. Approximately half of the participants described experiences of discrimination ranging from subtle mistrust to open rejection of treatment, primarily from patients. These experiences are consistent with narrative and scoping reviews documenting racism and discrimination as persistent features of healthcare systems, contributing to additional emotional burden for migrant professionals [19]- [20].

These experiences can be more fully understood through the concept of othering, which describes the social and symbolic processes through which individuals or groups are positioned as different from a perceived normative majority and thereby become marked as outsiders within institutional and interpersonal contexts [33]. Within the German healthcare system, such processes have been shown to contribute to the marginalization and silencing of people with migration backgrounds, including healthcare professionals, thereby reproducing existing structural inequalities [34]. Participants’ accounts suggest that othering operated not only through overtly discriminatory encounters but also through more subtle mechanisms, such as implicit cultural or religious insensitivities among colleagues regarding practices such as fasting during Ramadan. These findings echo research on the social integration of Middle Eastern physicians in Germany, which highlights both supportive professional relationships and experiences of marginalization [17].

Marked regional variation in the frequency and form of discriminatory encounters indicates that local institutional cultures and community contexts shape how othering unfolds in practice. In response to these experiences, participants developed pragmatic coping strategies, including ignoring minor incidents, redirecting conversations, involving supervisors, or adopting protective communication practices with patients. While these strategies reflect individual resilience and agency, reliance on individual adaptation risks obscuring the need for structural and organizational responses. As Yuval-Davis (2006) emphasizes, belonging and othering are not merely individual experiences but are produced and reproduced at the level of social, institutional and political structures (32). The findings therefore suggest that addressing racism and othering within the German healthcare system requires moving beyond individual coping toward institutional accountability, including clear anti-discrimination policies, intercultural training and systematic documentation of discriminatory incidents.

Taken together, the experiences of racism, coping strategies and cultural dynamics reported in this study reveal a complex negotiation between individual agency and structural marginalization. While participants actively navigated othering processes through pragmatic adaptation, the persistence of these experiences underscores the need for organizational-level strategies to promote equal professional recognition and belonging among migrant physicians.

### Intercultural competence and cultural brokerage

Cultural dynamics were predominantly framed as resources rather than obstacles. Shared language and cultural knowledge with Arab patients facilitated trust-building, nuanced interpretation of symptoms and effective communication, supporting previous findings that migrant physicians can enhance culturally responsive care [14]. At the same time, the visibility of cultural or religious practices occasionally exposed participants to misunderstanding or stereotyping, illustrating how intercultural competence operates simultaneously as a professional resource and as a dimension through which difference can become consequential within workplace interactions. The absence of institutional guidelines for managing cultural expectations further indicates a need for organizational-level strategies, including intercultural training and clear policies.

Participants’ accounts also highlighted their role as cultural and institutional mediators for patients with migration backgrounds. Shared linguistic and cultural knowledge enabled them to bridge communication and institutional gaps, explain healthcare procedures and support patients in navigating complex institutional structures. While this role may improve access to care and patient understanding, it often remains informal and largely unrecognized within organizational structures. This finding aligns with concepts of cultural brokerage in healthcare, whereby professionals with migration backgrounds facilitate communication and mutual understanding between institutions and culturally diverse populations [35]. However, reliance on informal individual mediation raises concerns about the sustainability of depending on migrant physicians as ad hoc interpreters and cultural guides, rather than systematically providing professional interpreting services and patient education.

Overall, the findings suggest that professional integration should be conceptualized as an institutional responsibility rather than primarily an individual adaptation task. Structural reforms addressing recognition procedures, onboarding, administrative support, language assistance and intercultural competence are likely to benefit not only migrant physicians but the healthcare workforce more broadly [16].

### Strengths and limitations

A major strength of this study is the in-depth exploration of experiences from the perspective of Arab physicians across multiple specialties and regions. The qualitative design enabled nuanced insights into institutional mechanisms and everyday practices. Limitations include the non-probabilistic sample and potential self-selection bias, which may limit transferability. Additionally, interviews were conducted in two languages and although translation procedures were implemented, subtle meaning shifts cannot be fully excluded. A further limitation concerns the use of telephone interviews for some participants. Although telephone interviews provided practical flexibility and facilitated participation across geographical locations, the absence of visual and contextual cues may have limited access to non-verbal expressions and aspects of the interaction that can contribute to rapport, probing and interpretation in qualitative interviews [36]- [37]. Consequently, some contextual or non-verbal nuances of participants’ experiences may not have been fully captured. Nevertheless, the same semi-structured interview guide and core thematic areas were used across interviews and follow-up questions and probes were employed to encourage elaboration and clarification. Future research should include comparative perspectives of German-trained physicians and institutional stakeholders to triangulate findings.

A further limitation concerns the interpretive nature of reflexive thematic analysis. The development and definition of themes necessarily involved researcher interpretation and alternative interpretations of participants’ accounts may therefore be possible. Although the analytic process involved iterative engagement with the data, examination of divergent cases and critical reflection on interpretive decisions, the five themes should be understood as an interpretive account of shared patterns of meaning rather than as objective or exhaustive representations of participants’ experiences. The findings should consequently be interpreted in light of the researchers’ positionality and the context-specific nature of the sample.

## Conclusion

Physicians with an Arab migration background constitute an essential component of the German healthcare workforce and play a crucial role in addressing ongoing physician shortages. The findings of this study demonstrate that professional integration is not merely an individual adaptation process but is strongly shaped by systemic and institutional conditions. While Germany offers robust structural prerequisites for high-quality medical practice, substantial barriers remain in the areas of licensure, administrative procedures, workplace integration and intercultural inclusion. The findings further highlight the often-overlooked role of migrant physicians as cultural and institutional mediators who support patients with migration backgrounds in navigating healthcare services, understanding institutional procedures and overcoming communication barriers. While this contribution can facilitate access to care and improve patient understanding, it frequently relies on informal individual efforts and remains insufficiently recognized within organizational structures. To strengthen professional integration and fully utilize the potential of migrant physicians, healthcare institutions and policymakers should implement coordinated support measures. These include more transparent and streamlined licensure procedures, structured onboarding and mentoring programs, improved administrative support, wider availability of professional interpreting services, systematic intercultural training and organizational strategies to prevent discrimination and promote equal professional recognition. Recognizing and supporting the mediating role of migrant physicians should also be considered part of broader institutional integration strategies. Addressing these issues is essential not only for supporting migrant physicians but also for enhancing the resilience, inclusiveness and long-term sustainability of the German healthcare system.

## Data Availability

All data supporting the findings of this study are available within the paper and its Supplementary Information.
